# Using Confocal Microscopy and Pigment Analyses to Detect Adverse Insecticide Effects in non-target Freshwater Diatom species - a proof-of-concept Using *Nitzschia palea*

**DOI:** 10.1007/s00128-023-03741-5

**Published:** 2023-06-07

**Authors:** Anrich Kock, Wihan Pheiffer, Victor Wepener, Nico J. Smit, Jonathan C. Taylor

**Affiliations:** 1 Water Research Group, Unit for Environmental Sciences and Management, North-West University, Private bag X6001, Potchefstroom, 2520 South Africa; 2 DSI/NWU Preclinical Drug Development Platform, North-West University, Private bag X6001, Potchefstroom, 2520 South Africa; 3 Unit for Environmental Sciences and Management, North-West University, Private bag X6001, Potchefstroom, 2520 South Africa; 4 South African Institute for Aquatic Bioaffiliationersity (SAIAB), Private Bag 1015, Grahamstown, 6140 South Africa; 5 North-West University, 11 Hoffman Street, Potchefstroom Campus, Building F20, Room 63, Potchefstroom, 2531, South Africa

**Keywords:** Diatoms, DDT, Deltamethrin, Mixture, Chlorophyll, Cell deformities

## Abstract

The persistence of insecticides in aquatic environments is a cause of concern and to date hardly any studies have focused on the effects that DDT and deltamethrin have on non-target freshwater diatom communities. The application of diatoms in ecotoxicological studies is well acknowledged and therefore this study used laboratory bioassays to determine the effects that DDT and deltamethrin have on a monoculture of a diatom indicator species, *Nitzschia palea*. The insecticides affected the morphology of chloroplasts at all exposure concentrations. These effects were a maximum reduction in chlorophyll concentrations (4.8% and 2.3%), cell viability (51% and 42%), and increases in cell deformities (3.6% and 1.6%) following exposure to DDT and deltamethrin respectively. Based on the results we propose that methods, such as confocal microscopy, chlorophyll-α analysis and cell deformities are useful tools in assessing the effects of insecticides on diatoms.

Malaria vector control pesticides, such as dichlorodiphenyltrichloroethane (DDT – an organochlorine pesticide) and deltamethrin (DTM – a pyrethroid pesticide), are still widely used in developing countries through indoor residual spraying (IRS) (Wolmarans et al. 2021). Even though DDT has been internationally banned, it’s use for human health purposes (malaria vector control) has been ongoing in South Africa since the 1990s (Bouwman et al. 2011). In KwaZulu-Natal (South Africa) both insecticides are sprayed as part of an IRS program for malaria vector control, with DDT) sprayed in traditional and DTM in western-type houses (Hargreaves et al. 2003) with mixtures of these insecticides entering the environment. The continued presence of DDT in aquatic ecosystems in Africa has been demonstrated with recent research confirming increased levels of DDT and their metabolites in freshwater amphibians and fishes (Gerber et al. 2016; Pheiffer et al. 2018; Wolmarans et al. 2021). DDT and its metabolites persist in the environment for long periods of time and due to its lipophilic nature, it bioaccumulates through the food web (Bouwman et al. 2011; Volschenk et al. 2019). Serada and Meinhardt (2005) reported high levels of DTM in water and sediments of water systems of KwaZulu-Natal where malaria vector control is carried out. Even though Wolmarans et al. (2020) identified DTM as one of the vector control pesticides with the highest toxicity potency, limited research has been conducted on the effects of this pesticide to non-target organisms. Although pesticides are advantageous to use, they have potential negative effects on the environment, human health, and wildlife, especially on non-target organisms (Margni et al. 2002). There is a paucity of research on the primary and secondary effects that these pesticides have on non-target organisms (Wolmarans et al. 2022), including primary producers such as diatoms. Recent microcosm studies by Kock et al. (2022) demonstrated that low levels of DDT, DTM and their mixtures resulted in changes in diatom community traits and decreased vitality in several diatom species.

Insecticide uptake by diatoms takes place through different pathways. Lipophilic DDT binds to the cell’s lipid-protein membranes by attaching to the photosynthetic mechanisms of the membranes (Macfarlane et al., 1972). Deltamethrin on the other hand is highly water soluble. Subsequently the diatoms take up deltamethrin through osmotic processes where it accumulates, resulting in toxic effects, as was observed for *Skeletonema costatum* (Baeza-Squiban et al. 1987). Only one previous study on the effects of DDT on freshwater *Nitzschia* sp. and an unidentified diatom species was reported (Miyazaki and Thorsteinson 1972), with no published studies on the effects of DTM to diatoms.

A sensitive and rapid method to assess the toxicity of chemicals on diatoms is the measurement of their fluorescence. Due to the pigments of phototrophic organisms, auto florescence analysis can be carried out (Neu and Lawrence 1997). Earlier studies made use of chlorophyll fluorescent-based assays to test the effects of pollutants on freshwater microalgae (Choi et al. 2012) and phytoplankton (Seguin et al. 2002). One such method includes the use of confocal laser scanning microscopy (CLSM) (Fricke et al. 2017). This method has the advantage of visually and quantitatively determining the *in-situ* fluorescent properties of single celled organisms. The aim of this study was to use CLSM to determine the effects of DDT and DTM and their mixtures on the chloroplast of a diatom indicator species, *Nitzschia palea* (Kützing) W. Smith. The hypothesis tested was that DDT and DTM would inhibit the photosystems of the diatom cells, thereby negatively affecting their vitality as reflected by decreased chlorophyll-α concentrations.

## Materials and methods

*Nitzschia palea* (Kützing) W. Smith was chosen as indicator species. According to Taylor et al. (2007), N. *palea* is a cosmopolitan species that is found in heavily polluted and eutrophic water. A long-standing and well-established laboratory monoculture (Algal Research Group at NWU) of *N. palea* was grown in a 50% GBG 11 culture medium (Zhang and Lian 2020) with a 50% culture replacement weekly. Cultures were allowed to acclimate and colonise for two weeks before exposures. All cultures (50,000 cells per mL) were grown at 24ºC, under permanent 27,000 K light regime in a controlled culture room. The cultures were treated with 3 µl rhodamine-123 dye (0.1 N) (final culture medium concentration of 1µM) to ensure cell wall (frustule) fluorescence. Rhodamine-123 was specifically used as it does not affect the functionality or vitality of diatoms (Kucki & Fuhrmann-Lieker, 2011). The cultures (30 ml) together with 200 ml BG 11 culture media were added into 250 ml Erlenmeyer flasks. After 14 d the treatments (three replicates per treatment) were exposed to a single dose of either technical grade T-DDT (Dr, Ehrenstorfer GmbH), commercial grade C-DDT (AVI-DDT 750, Avima (Pty) Ltd), deltamethrin (Decatix® 3, Coopers), and a mixture (1:1) of C-DDT and DTM, each at a high (H) and low (L) concentrations. Commercial-grade DDT was selected for the mixture experiments since the DTM was also a commercial grade pesticide and the mixture toxicity would thus reflect exposure realism. The selected exposure concentrations and application scenario were the same used by Kock et al. (2022) in microcosm experiments to assess the effects of DDT, DTM and their mixture on diatom community structures, i.e., a single application at the onset of the assay and the following responses over a 28 d period. Once-off exposure concentrations were: DDT [358 $\mu$g/L (H) and 35.8 $\mu$g/L (L)] and DTM [1.9 $\mu$g/L (H) and 0.19 $\mu$g/L (L)]. Once-off exposure concentrations were; DDT [358 µg/L (H) and 35.8 µg/L (L)] and DTM [1.9 µg/L (H) and 0.19 µg/L (L)]. The pesticide concentration in all stock solutions were analytically confirmed using standard single-phase liquid-liquid extraction and GC-ECD analytical techniques and using PCB143 as internal standard (Kock et al. 2022). The average recovery rates for DDT and DTM in the different treatments ranged between 60 and 61% (Table S1). Untreated and solvent controls were included. Triplicate samples from each treatment were collected at 96 h, 14 d and 28 d time intervals after the initial single dose exposure for analysis.

The viability of *N. palea* was determined based on the chlorophyll-α content as described by Swanepoel et al. (2008). Briefly, 3 ml of each replicate sample was filtered through a Whatman glass filter and 10 ml of ethanol (95%) was added and incubated at (78 °C) for 5 min. Samples were allowed to cool to room temperature (in the dark), transferred to cleaned cuvettes and absorbance was determined in triplicate at 665 and 750 nm. Three drops of hydrochloric acid (0.3 M) were added to each sample to convert chlorophyll-*α* to phaeophytin-*α* and absorbances were again determined at the same wavelengths. The total chlorophyll-*α* was calculated according to the following equation:

$$Chlorophyll - \alpha \left( {\mu g/l} \right){\text{ }} = {\text{ }}\left[ {\left( {A665 - A750} \right) - \left( {A665a - A750a} \right)} \right]{\text{ }}x{\text{ }}28.66{\text{ }}x{\text{ }}VeVm$$

Where: A665 and A750 are the absorbances at 665 and 750 nm, A665a and A750a are the absorbances after acidification, 28.66 is the ethanol absorption coefficient; Ve is the volume of ethanol (ml), and Vm the volume of sample filtered (ml). The percentage live cells were calculated relative to the control for each treatment. Percentage live cells (%) = CeCc×100; where: Ce is the Chlorophyll-*α* concentration of the sample, and Cc is the Chlorophyll-α concentration of the control.

After the 96 h, 14 d and 28 d exposure periods, a diatom sample from each treatment was washed with distilled water to ensure that there was no residual dye that could cause background interference. A minimum of 15 confocal images (n = 15) were taken for each exposure treatment and time period over the 28-d exposure period using a Nikon D-Eclipse C1 CLSM with an x 60 1.4 NA ApoPlanar oil objective. The microscope was equipped with red Helium/Neon (505 nm/565 nm) and green Krypton (488 nm/515 nm) Spectra-physics lasers. The lasers were used to excite and detect chloroplasts (red) and the frustules with the absorbed rhodamine-123 dye (green). A medium pinhole together with a 3 µs/scan speed was used. All CLSM images were acquired using identical settings on Nikon EZ 200 software. Fluorescence intensities of specimens acquired with the CLSM were quantified using ImageJ and mean corrected total cell fluorescence (CTCF) analysed (Schneider et al. 2012).

Permanent slides of the 28 days exposure samples were prepared post-CLSM analysis. This was done to determine if any frustule deformities occurred from the exposure. Slide preparation was done according to Taylor et al. (2005) and viewed under a Nikon 80i compound microscope with an x100 1.4 N.A. oil immersion objective. A total of 100 frustules were counted and the percentage of deformed frustules calculated. Cell deformities were based on morphological characteristics, including cell shape and symmetry.

To determine significant differences (p < 0.05) between pesticide treatments and exposure period and joint effect of the two factors, a two-way analysis of variance (ANOVA) and multiple pair-wise tests were conducted. Prior to conducting the ANOVA tests, data were tested for normality (Shapiro-Wilk test) and homogeneity of variance (Levene’s test) (Zar 1996). If data did not meet the parametric assumptions, they were log_10_ transformed prior to analysis. Significant differences (p < 0.05) between percentage live cells for each exposure was determined by means of a two-way ANOVA with Tukey’s multiple comparisons *post hoc* test. All statistical analyses and graphs were produced using GraphPad Prism version 9.

## Results

**Fig. 1:**
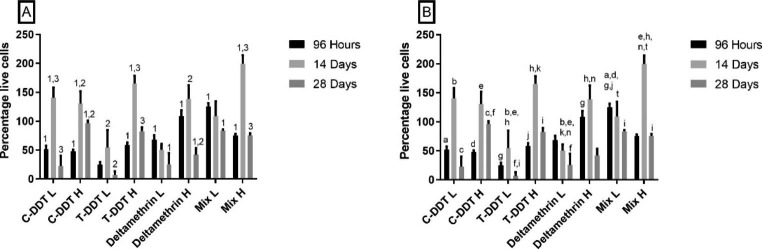
Percentage live cells (mean ± SEM) of *Nitzschia palea* after 96 h, 14 days, and 28 days exposures to DDT, deltamethrin, and Mix (DDT: deltamethrin). All percentage live cells are relative to the control samples. (A) Means of columns representing different pesticide exposure treatments with common numerals indicating significant differences between exposure periods. (B) Means of columns between exposure treatments with common letters indicating significant differences between insecticide exposure treatments. C – Commercial grade, T – Technical grade, L – Low concentration, H – High concentration

Significant increases (p < 0.05) in percentage live cells (based on chlorophyll-α concentrations) from 96 h to 14 d was noted in all treatments, except DTM L and Mix L, thereafter there was a decrease until the end of the exposure period (Fig. 1). Deltamethrin L and Mix L showed a decrease in percentage live cells after each exposure period, with a significant (p < 0.05) decrease between 96 h and 28 d (Fig. 1). Significant differences were observed between insecticide concentrations (H vs. L) (F = 47.22, p < 0.0001) and between exposure periods (F = 10.41, p = 0.0260). A significant interaction between the insecticide concentration and exposure period was also observed (F = 11.15, p < 0.0001).

**Fig. 2:**
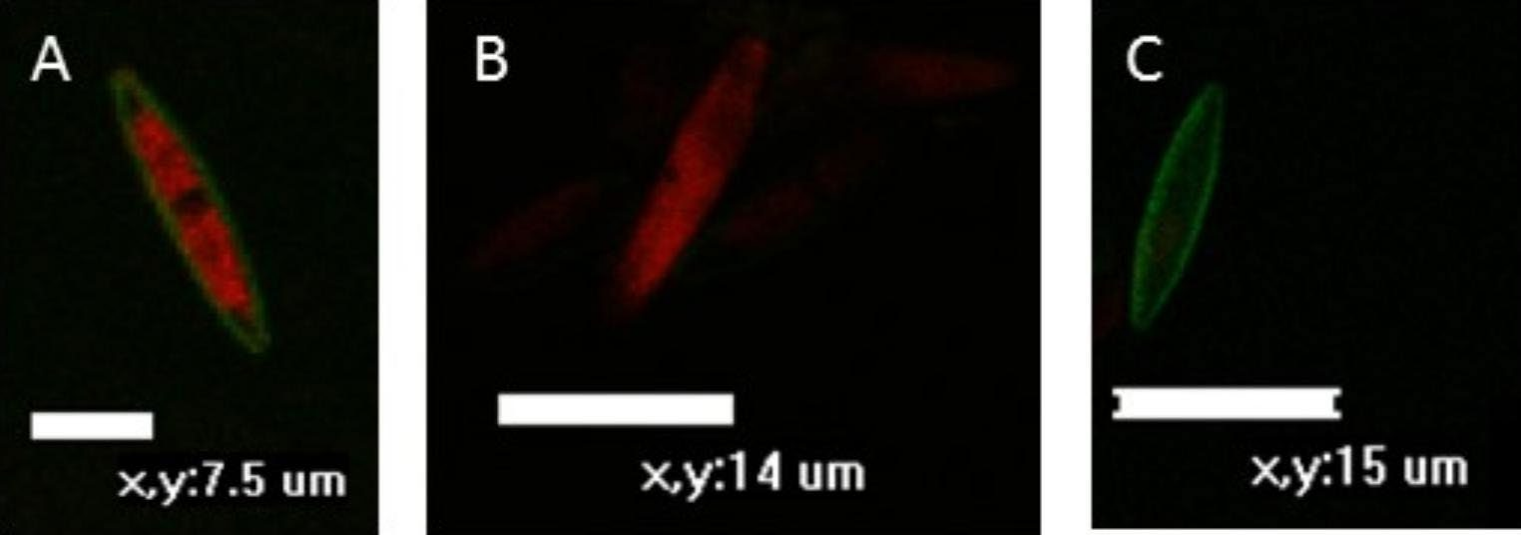
Confocal laser scanning microscopy images showing *Nitzschia palea* as a healthy cell **(A)**, and reactions after exposure to insecticides; frustule dispersion and a burst chloroplast **(B)** and a dead cell with no chloroplast present **(C)**

**Fig. 3:**
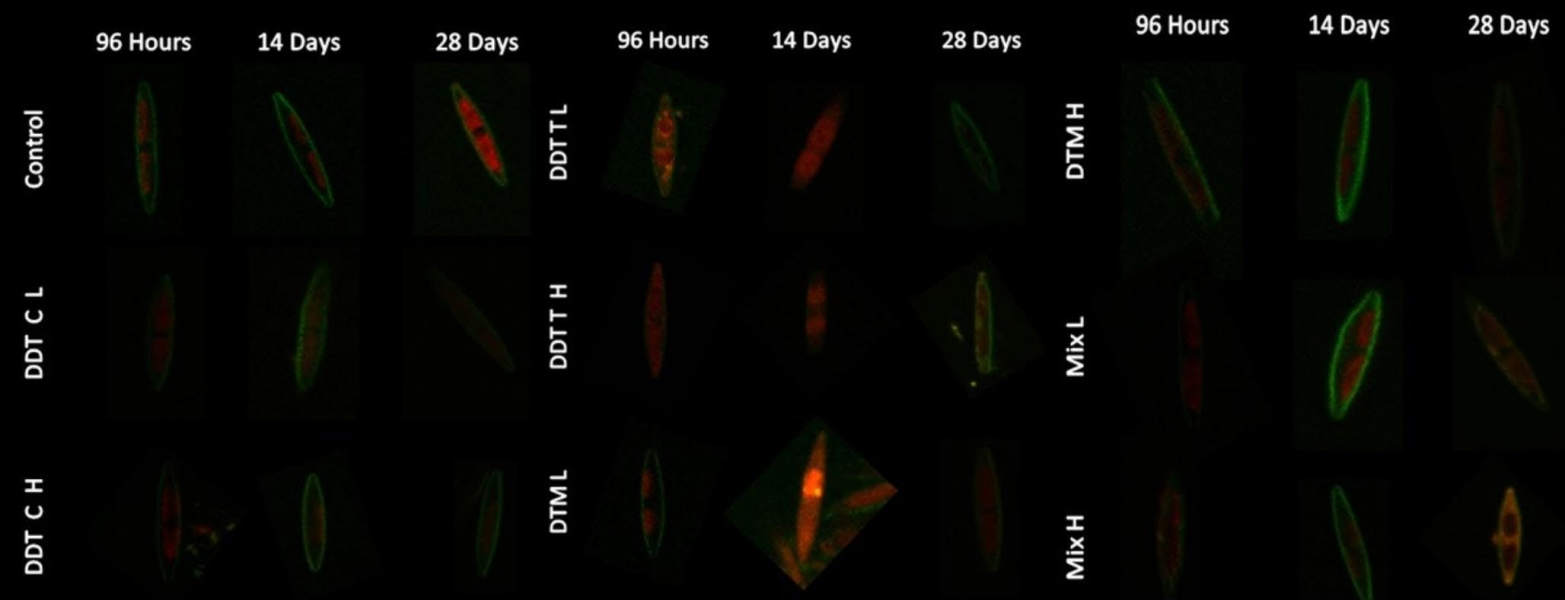
Confocal laser scanning microscopy images of the *Nitzschia palea* diatoms exposed to the different insecticides over a time period of 96 h, 14 days and 28 days. C – Commercial grade, T – Technical grade, L – Low concentration, H – High concentration

Green fluorescence in the confocal images indicates the diatom frustule and red fluorescence of the chloroplasts. For the control, consistent healthy cells were present with the frustule clearly visible, as well as the two distinct regions containing the chloroplasts (Fig. 2A). Burst chloroplast (chloroplast with no true shape occupying the entire cell) where noted in the exposure treatments showing chlorophyll spread throughout the cell (Fig. 2B). Additionally, the absence of intact frustules (Fig. 2B) and the absence of chloroplasts (Fig. 2C), or deformed or shrunken chloroplasts were recorded (Fig. 3 – C-DDT H, 14 d). However, there was no clear trend on how *N. palea* reacted to the different insecticides over time. Within the same treatments and exposure periods there were diatoms showing several of the above-mentioned responses.

Corrected total cell fluorescence (CTCF) (from the 15 confocal images, multiple diatoms per image) showed that diatoms exposed to the insecticides had a lower fluorescent cell intensity compared to the control over all exposure times (Figure S1). There were significant differences (p < 0.05) between all the exposed treatments and the control over 96 h and 14 d (Figure S1). Significant differences were observed between insecticide concentrations (F = 23.76, p < 0.0001) and between exposure periods (F = 24.38, p < 0.0001), also between the insecticide concentration and exposure period (F = 6.988, p < 0.0001). Only the control had no deformed cells. The highest percentage deformities were recorded for the DDT treatments (i.e. between 3 and 4%) with DTM causing less deformities, 1% and 2% in DTM L and DTM H respectively. The mixtures displayed a similar percentage deformity to the individual DDT exposures resulting in 3% and 4% deformities in the Mix L and Mix H treatments, respectively.

## Discussion

Information on the physiological status of the diatoms can be obtained from chlorophyll analysis as it is coupled to numerous biochemical processes as well as to the photosynthetic electron transfer chain. Fluorescence of chlorophyll-α has successfully been used as an indicator of the effects of pollutants such as pesticides on photosynthesis of phytoplankton (Seguin et al. 2002; Choi et al. 2012). The confocal imaging applied during this study clearly showed that the insecticide exposures degraded both the chloroplast and the chlorophyll-α within the cell and only left the cell frustule intact. The damage as manifested by a decrease in chlorophyll-α intensity could be due to damage of the chloroplast structure or a reduction in or loss of chloroplasts resulting in a reduction in the photosynthetic output of the cells. Both DDT (Lee et al. 1976) and DTM (Bader and Schüler 1996) are known to inhibit cell electron transport reactions. The difference is that DDT inhibits cyclic related phosphorylation at photosystem I (PSI) (Lee et al. 1976), while DTM inhibits photosystem II (PSII) (Bader and Schüler 1996). Deltamethrin’s strong phototoxic inhibition of the photosynthetic electron transport reactions is owed to the two bromides on the halogen side of the molecule and is considered the stronger inhibitor compared to other pyrethroids (Bader and Schüler 1996). This results in DDT causing a stoppage in the production of NADPH to fuel the reaction during PSII. The inhibition of PSII by deltamethrin results in a shutdown of photosynthesis within the cell. These effects are shown as an immediate decrease in percentage live cells for DTM L, and a delayed effect for DDT exposures.

Reduced photosynthetic activity can lead to a reduction in carbon fixation and subsequent reduction of chlorophyll-α concentrations (Macfarlane et al., 1972) and ultimately the interruption of energy production needed for cell growth (UCIPM, 2019). In the current study the cell viability (chlorophyll-α concentration) in the DTM L and Mix L exposures showed a steady decrease from the onset of the experiment and persisted over the entire exposure period. The culture was not able to stabilise following the initial exposure and subsequent toxic effect, and the population therefore could not recover. The continuous decrease in viable cells ultimately resulted in the culture crashing. Similar decreases in photosynthetic processes were found when periphyton communities were exposed to glyphosate (Smedbol et al. 2018) and 2,3 glyphosate single and mixture treatments (Lozano et al. 2018).

Macfarlane et al. (1972) found that DDT reduced and changed the shape of the chloroplast in diatom cells. Fidalgo et al. (1993) also reported that there was a reduction in chloroplast volume after exposure to DTM. This destruction of lipid and protein membrane structures is attributed to a sequence of reactions caused by highly reactive molecules formed due to inhibition of PSII (UCIPM, 2019). Membrane disruption results in leakage causing rapid disintegration and drying of the cell and cell organelles (UCIPM, 2019). This was visualised in the confocal images, where either the chloroplast was reduced in size, leaked (burst) inside the frustule and the absence of an intact frustule.

In contrast to the control sample, morphological (shape and symmetry) cell deformities were observed for all the treatments. *Nitzschia palea* diatom cultures are regarded as a “tolerant” species that can withstand extreme conditions, including heavily polluted waters, extreme nutrient loading, and temperature variation (Taylor et al. 2007). Thus, the relatively low percentage of cell deformities recorded for *N. palea* during this study can be considered as meaningful and demonstrates that the insecticides disturbed cell wall synthesis thus changing the morphology of cells in the exposed cultures. Similar morphological changes were found in *Nitzschia delicatissima*, following exposure to DDT (Macfarlane et al., 1972). To place the percentage deformities observed during this study in context, Lavoie et al. (2017) reported 0.5–2% deformed cells from highly polluted sites contaminated by high metal concentrations together with organic contamination, pesticides and increased pH).

This study demonstrated the successful application of the cosmopolitan diatom species, *N. palea*, as well CLSM techniques to demonstrate the responses of chloroplast photosynthetic efficiency, as a bioindicator. Importantly these data indicate that the effects of commonly used pesticides and their mixtures have significant negative effects on non-target primary producers. This highlights the negative effects to primary production and ecosystem functioning, which is too often neglected when assessing ecosystem responses to insecticide exposure.
